# Stratifying the risk of NAFLD in patients with HIV under combination antiretroviral therapy (cART)

**DOI:** 10.1016/j.eclinm.2021.101116

**Published:** 2021-09-05

**Authors:** Jenny Bischoff, Wenyi Gu, Carolynne Schwarze-Zander, Christoph Boesecke, Jan-Christian Wasmuth, Kathrin van Bremen, Leona Dold, Jürgen K Rockstroh, Jonel Trebicka

**Affiliations:** a Department of Internal Medicine I, Venusberg Campus 1, University Hospital Bonn, 53127 Bonn Germany,; b Department of Internal Medicine I, University Hospital Frankfurt, Germany; c German Center for Infection Research (DZIF), partner site Cologne-Bonn, Bonn, Germany

**Keywords:** Steatosis, Hiv, Nafld, Cap, cART, AST, aspartate aminotransferase, APRI, AST to platelet ratio index, BMI, body mass index, CAP, controlled attenuation parameter, ART, antiretroviral treatment, DAA, direct-acting antiviral, FAST, FibroScan-AST, FIB4, fibrosis-4, HCV, chronic hepatitis C, INSTI, integrase strand transfer inhibitors, NAFLD, Non-alcoholic fatty liver disease, NASH, non-alcoholic steatohepatitis, PrEP, pre-exposure prophylaxis, PLHIV, people living with HIV, TAF, tenofovir-alafenamid, TDF, Tenofovir disoproxilfumarate, TE, transient elastography

## Abstract

**Background:**

De novo steatosis is the main criteria for non-alcoholic fatty liver disease (NAFLD), which is becoming a clinically relevant comorbidity in HIV-infected patients. This may be due to the HIV virus itself, as well as long-term toxicities deriving from antiretroviral therapy. Therefore, HIV infected patients require prevention and monitoring regarding NAFLD.

**Methods:**

This study investigated the differential role of body mass index (BMI) and combination antiretroviral treatment (cART) drugs on NAFLD progression. This single center prospective longitudinal observational study enrolled HIV monoinfected individuals between August 2013 to December 2018 with yearly visits. Each visit included liver stiffness and steatosis [defined as controlled attenuation parameter (CAP)>237 dB/m] assessment by annually transient elastography using an M- or XL-probe of FibroScan, and calculation of the novel FibroScan-AST (FAST) score. Risk factors for denovo/progressed steatosis and tripling of FAST-score increase were investigated using Cox regression model with time-dependent covariates.

**Findings:**

319 monoinfected HIV positive patients with at least two visits were included into the study, of which 301 patients had at least two valid CAP measurements. 51·5%(155) patients did not have steatosis at first assessment, of which 45%(69) developed steatosis during follow-up. A BMI>23 kg/m^2^ (OR: 4·238, 95% CI: 2·078–8·938; *p* < 0·0001), tenofovir-alafenamid (TAF) (OR: 5·073, 95% CI: 2·362–10·899); *p* < 0·0001) and integrase strand transfer inhibitors (INSTI) (OR: 2·354, 95% CI: 1·370–4·048; *p* = 0·002), as well as type 2 diabetes mellitus (OR: 7·605, 95% CI: 2·315–24·981; *p* < 0·0001) were independent predictors of de novo steatosis in multivariable analysis. Tenofovir disoproxilfumarate (TDF) was associated with a lower risk for weight gain and steatosis progression/onset using CAP value (HR: 0·28, 95% CI: 0·12–0·64; *p* = 0·003) and FAST scores (HR: 0·31, 95% CI: 0·101–0·945; *p* = 0·04).

**Interpretation:**

Steatosis can develop despite non-obese BMI in patients with HIV monoinfection under cART, especially in male patients with BMI over 23 kg/m^2^. While TAF and INSTI increase the risk of progression of steatosis, TDF was found to be independently associated with a lower risk of a clinically significant weight gain and thereby, might slow down development and progression of steatosis.

**Funding:**

There was no additional funding received for this project. All funders mentioned in the ‘declaration of interests’ section had no influence on study design, data collection and analysis, decision to publish, or preparation of the manuscript.

**Research in context**

**Evidence before this study**

We searched Pubmed for key publications investigating the relationship between the treatment of HIV and steatosis using the search terms ‘non-alcoholic fatty liver disease’ ‘steatosis’, ‘cART’ and ‘HIV’. The prevalence of Non-alcoholic fatty liver disease (NAFLD) is estimated to be between 13%−73% in HIV positive patients. In addition to common known risk factors such as metabolic syndrome, insulin resistance and diabetes mellitus, further HIV specific risks have been described, but the relationship between individual antiretroviral drugs for HIV therapy and hepatic steatosis risk has not yet been well established.

**Added value of this study**

In our prospective longitudinal study with annual visits up to five years, the individual development and progression of hepatic steatosis was assessed. HIV patients showed progredient development and progression of steatosis despite non-obese BMI. Furthermore, while tenofovir disoproxilfumarate treatment was protective, integrase strand transfer inhibitors and tenofovir-alafenamid induced development and progression of steatosis probably by boosting weight gain. Finally, the Fibroscan-AST score is suitable to assess the liver damage of HIV patients.

**Implications of all the available evidence**

Development and progression of hepatic steatosis in HIV patients needs to be monitored regularly. The recently developed Fibroscan-AST score may be an appropriate screening tool in this population.

Alt-text: Unlabelled box

## 1 Introduction

Improvement in HIV treatments is leading to significant shifts in the focus of HIV patients clinical care. In the pre-antiretroviral treatment (pre-ART) era, AIDS-defining diseases were among the greatest threats. Now that modern antiretroviral combination therapy allows persistent control of HIV replication and has led to a near-to-normal life expectancy, the focus of HIV caregivers is on the management of non-AIDS defining comorbidities including non-alcoholic fatty liver disease (NAFLD) and the metabolic syndrome in an aging HIV-population.

Prevalence of NAFLD in the general population is well documented and estimated to be around 25% and is related to the occurrence of a metabolic syndrome. Interestingly, in HIV patients, NAFLD prevalence ranges between 13% to 73% [1], [2], [3], [4], [5], [6], [7], [8], [9]. Specific HIV related risk factors (e.g. persistent immune activation and antiretroviral drugs) may play an additional role and result in an altered course of liver disease [10,11]. Yet, NAFLD has been described even in lean HIV monoinfected individuals [12], which raises the question of surveillance of liver phenotype and associated risk factors [13,14]. Transient elastography (TE) and controlled attenuation parameter (CAP) are validated as non-invasive tests for hepatic fibrosis and steatosis with high diagnostic accuracy [15,16]. More recently, an additional non-invasive score, the FibroScan-AST (FAST-) score has been developed, which may be useful for detection of progressive non-alcoholic steatosis (NASH) and surveillance for steatosis in HIV patients [17].

Modern combined ART (cART) supresses viral replication and prevents AIDS-defining diseases such as wasting syndrome. Therefore, weight changes shortly after initiation of ART have to be considered as a return-to-health phenomenon. Indeed, weight gain after starting antiretroviral therapy has been associated with improved survival in HIV-patients particularly in those with more advanced disease stages [18,19]. However, pronounced effects of integrase strand transfer inhibitors (INSTI) as well as tenofovir alafenamide (TAF) on body weight and BMI have been reported lately in people living with HIV (PLHIV) [20], [21], [22], [23]. Whether these effects are associated with a higher risk of developing hepatic steatosis has not been well described so far. Therefore, we performed a longitudinal prospective observational study to screen for development or progression of steatosis as a hallmark of NAFLD and identify related risk factors for the emergence of hepatic steatosis in PLHIV.

## 2 Methods

### 2.1 Study design and study cohort

We conducted a single center longitudinal prospective observational study enrolling HIV positive patients, which presented at the HIV outpatient clinic of University Hospital Bonn. Enrolment started on 1st of August 2013 and was completed 24 months afterwards. Patients were followed annually at the outpatient clinic and the last visit to be considered in this analysis had to be on or before 31th of December 2018. The study was carried out in accordance with the Declaration of Helsinki and was approved by the institution's human research committee (279/14, 2014, 2016 and 2019).

Eligibility criteria comprised an age >18 years, a confirmed HIV diagnosis and a self-reported alcohol intake of less than 30 g/day for male participants and 20 g/day for female participants. A written informed consent had to be signed. Patients with concomitant viral hepatitis B (HBV) or C (HCV) coinfection were excluded.

Baseline characteristics including age, gender, ethnicity, BMI and comorbidities were recorded initially. Clinical chemistry comprised platelet count, transaminases, liver function test, metabolic markers, creatinine and C reactive protein (CRP). Mode of HIV transmission, estimated or known duration of infection, time and type of antiretroviral treatment, CD4 and viral load were assessed for HIV infection.

### 2.2 Hepatic steatosis evaluation

Liver stiffness (LS in kPa) and steatosis (CAP value, expressed as dB/m) were assessed by TE at each visit using an M-Probe or XL-Probe of FibroScan® (Echosens, Paris, France. LS and CAP values were expressed as the median of all valid measurements. To ensure reliability, at least 10 times of the valid measurements were acquired, and the automatically calculated interquartile range (IQR) to median value ratio was less than 30% (IQR/median value ≤ 0·3). A success rate of at least 60% (calculated as the percentage of valid measurements out of the total number of measurements) had to be available to calculate LSM and CAP values [24]. While liver biopsy is considered the gold standard for diagnosing NAFLD, introduction of various non-invasive tools has led to the gradual abandonment of biopsies in studies in the interest of patient safety. For the classification of steatosis, we followed previous evaluations in HIV mono-infected patients, which have been used in several studies, although they might slightly overestimate prevalence of steatosis compared to MRI based evaluation. [2,25,26]

Cut-off values to define steatosis were set as follows: no signs of steatosis (S0) < 238 dB/m, beginning steatosis (S1): 238–259 dB/m, advanced steatosis (S2): 260–291 dB/m and severe steatosis (S3): >292 dB/m. Patients with no steatosis at the first assessment but that developed it in the follow-up were considered as de novo steatosis. Those patients with lower stage of steatosis at the first assessment but progressed to a higher stage were considered as steatosis progression. Significant liver fibrosis was determined with liver stiffness values ranging from 7·1–12·4 kPa or a fibrosis-4 (FIB4) score of 1·46–3·25 or an aspartate aminotransferase (AST) to platelet ratio index (APRI) score of 0·6 to 1·5. Severe fibrosis was diagnosed in cases of a liver stiffness above 12·5kPA or a FIB-4 above 3·26 or an APRI Score above 1·6.

All measurements were performed by experienced operators working in our out-patient clinic. Operators, 5 in total, were not blinded to the clinical details of the patients as they were in most cases involved in the routine care of our patients. To reduce an operator- associated bias a strict protocol and instructions of the manufacturer were followed performing the measurements.

FAST scores as defined by Newsome et al. were evaluated for each visit. NASH + NAS >4 + *F*>2 was ruled out with a FAST 〈 0·35 and ruled in with FAST 〉 0·67 [17]. A FAST score between 0·35 and 0·67 was considered as a gray area. FAST score significantly increase was defined as a tripling increase from the first assessment. [17]

### 2.3 Definition of subgroups and study outcomes

We hypothesize that there is an induction of weight gain following INSTI treatment and a weight stabilizing effect of tenofovir disoproxilfumarate (TDF), in contrast to a weight increasing effect due to TAF. The exposure visit point was defined as the visit switching to TAF, TDF or INSTI regimen, or the same as baseline visit if no such switch occurred. Therefore, we subdivided our HIV monoinfected cohort with >2 visits (*n* = 319) with regard to their treatment into groups containing either INSTI or TDF or TAF and compared each group to those not being administered to the respective drugs.

All participating patients were followed on an annual basis from 2013 to 2018 with valid CAP values and a FAST score for at least 2 years, up to 5 years or lost to follow-up. Primary endpoints were de novo development or progression of hepatic steatosis, FAST score of tripling increase and clinically significant weight gain, defined as a weight gain > 5% and >10% over the study period, respectively.

### 2.4 Statistical analysis

Continuous variables were summarized as mean ± standard deviation, categorical variables were showed as number and percentage. Comparisons of study cohort characteristics between different groups were performed using 2-sided t-tests and nonparametric Mann-Whitney-U test for continuous variables where eligible, and Chi-squared tests or Fisher's exact test for categorical data. For comparisons of continuous variables of two or more independent samples a one-way analysis of variance (ANOVA) for parameters normally distributed, otherwise, non-parametric Kruskal Wallis Test was performed. The repeated measurements between the first and the last visit, or between exposure visit and the last visit points were compared using a paired t-test.

Logistic regression was used to for model establishment and decision tree of de novo steatosis development. To furtherly establish a tool to stratify the de novo steatosis development risk, a decision tree was developed in 142 patients without steatosis at the first assessment of valid CAP value. The optimal cut-off values were decided for independent risk factor with the best Youden index. For internal validation, a 1000-time bootstrapping was performed to get the mean mortality rate with 95% confidence interval for the three different risk groups of de novo steatosis.

Cox regression with time-dependent covariates was used for independent risk factors of weight gain, FAST score of tripling increase and de novo steatosis development or progression, using the variables of each visit point. The parameters significant in univariate analysis were selected into multivariate analysis. The selection of independent risk factors was based on stepwise forward procedure with p-in <0·05. In the Cox regression model with time-dependent covariates, missing measures were imputed with last observation carried forward. The number of valid data of each variable in the univariate analysis are summarized in Supplementary Table 1 and Supplementary Table 2. Results of the univariate analysis of underlying comorbidity conditions that could influence the progress of NAFLD are listed in supplementary Table 3.

Differences were considered significant at *p*<0·05. Statistical analysis was performed using SPSS 25.0 (IBM©, USA), SAS Institute (Cary, USA) and R software version 3.6.1 (R core team, Vienna, Austria).

The manuscript was prepared in strict adherence to the STROBE guidelines for cohort studies.

### 2.5 Role of funding sources

All funders mentioned in the ‘declaration of interests’ section had no influence on study design, data collection and analysis, decision to publish, or preparation of the manuscript.

## 3 Results

### 3.1 Study cohort

Overall, as shown in Table 1, 319 HIV patients were included in our analysis. 77% (247) were male and the mean age and standard deviation at baseline was 45·7 ± 11·5 years. 76·2% (243) were of Caucasian origin and 16·3% (52) and 6·6% (21) of African and Asian origin, respectively. Mean follow-up time was 41·8 ± 14·8 months. Concomitant diabetes mellitus and/or arterial hypertension was present in 6·0% (19) and 20·4% (65) of patients at baseline, respectively. Most frequent routes of transmission were men who have sex with men (MSM) [55·2% (176)] and heterosexual contacts [21·6% (69)]. Mean time since HIV diagnosis was 9·4 ± 6·3 years and patients were on ART for 8·0 ± 5·9 years. 45·3% of patients had a nadir CD-4 cell count <200/µl and 27·2% were diagnosed as CDC category C.

**Table 1:** Comparison of baseline characteristics and last visit study population.

Parameter	Baseline (*n* = 319)	Last Visit (*n* = 319)	*P* value
Time of follow-up, month	··	41·8 ± 14·8	··
Male	247 (77·4)	··	··
BMI, kg/m^2^	24·9 ± 4·5	26·1 ± 6·2	<0·001
Weight, kg Weight gain, kg	77·2 ± 14·2	80·8 ± 20·7 3·8 ± 3·8	<0·001 ··
HIV RNA undetectable	251 (86·3)	269 (90·9)	<0·001
**cART**			<0·001
NRTI	294 (92·2)	279 (89·0)	
NNRTI	128 (40.0)	117 (37·4)	
PI/r	130 (41.0)	80 (25·6)	
INSTI	61 (19.0)	127 (40·6)	
Entry-inhibitor	3 (0·9)	2 (0·6)	
**Elastography and non-invasive test**			
Steatosis (CAP, dB/m)	234·6 ± 57·4	253·7 ± 62·0	0·003
S0 (< 237)	131 (54·6)	129 (41·0)	
S1 (238 – 259)	34 (14·2)	43 (13·7)	
S2 (260 – 291)	33 (13·8)	61 (19·4)	
S3 (>292)	42 (17·5)	82 (26·0)	
Liver stiffness, kPa	5·1 ± 2·2	5·5 ± 0·6	0·048
FIB-4 score	0·9 ± 0·5	1·2 ± 0·7	<0·001
APRI score	0·3 ± 0·2	0·3 ± 0·2	0·001
FAST score	0.1 ± 0·1	0.3 ± 0·2	<0·001
**Laboratory test**			
CD 4,%	29·8 ± 10	31·3 ± 10·1	<0·001
CD 8,%	42·9 ± 12·2	41·0 ± 11·6	<0·001
Cholesterol, mg/dl	197·6 ± 43·1	203·7 ± 42·8	0·007
HDL, mg/dl	49·4 ± 18·0	54·0 ± 18·3	<0·001
LDL, mg/dl	121·4 ± 31·7	132·5 ± 67·2	0·006
Triglyceride, mg/dl	187·6 ± 148·3	177·8 ± 134·0	0·516
AST, U/I	24·5 ± 14·2	28·8 ± 17·4	0·001
ALT, U/l	35·4 ± 18·1	32·7 ± 22·1	0·096
yGT, U/l	57·7 ± 54·8	46·6 ± 66·6	<0·001
Total Bilirubin, mg/dl	0·6 ± 0·5	0·6 ± 0·6	0·952
Creatinine, mg/dl	0·9 ± 0·2	0·9 ± 0·2	0·208
Platelets, G/l	225·8 ± 59·1	228·6 ± 57·6	0·740
Albumin, g/dL	44·1 ± 3·9	45·6 ± 5·2	<0·001
Glucose, mmol/l	95·2 ± 29·4	101·3 ± 33·6	<0·001
HBA1c,%	5·5 ± 0·9	5·6 ± 1·0	0·056
CRP, mg/L	3·9 ± 5·4	4·4 ± 9·7	0·354

P values were obtained using paired *t*-test or Chi-square test where appropriate.

Abbreviations: ALT, alanine aminotransferase; APRI, AST to platelet ratio index; AST, aspartate aminotransferase; BMI, body mass index; CAP, controlled attenuation parameter; cART, antiretroviral therapy; CRP, C reactive protein; FAST, FibroScan-AST; FIB-4, Fibrosis-4; HBA1c, hemoglobin A1c; HDL, high-density lipoprotein; INSTI, integrase strand transfer inhibitor; LDL, low-density lipoprotein; NNRTI, non-nucleoside reverse transcriptase inhibitor; NRTI, nucleoside reverse transcriptase inhibitor; PI/r, ritonavir-boosted protease inhibitor.

**Fig. 1:**
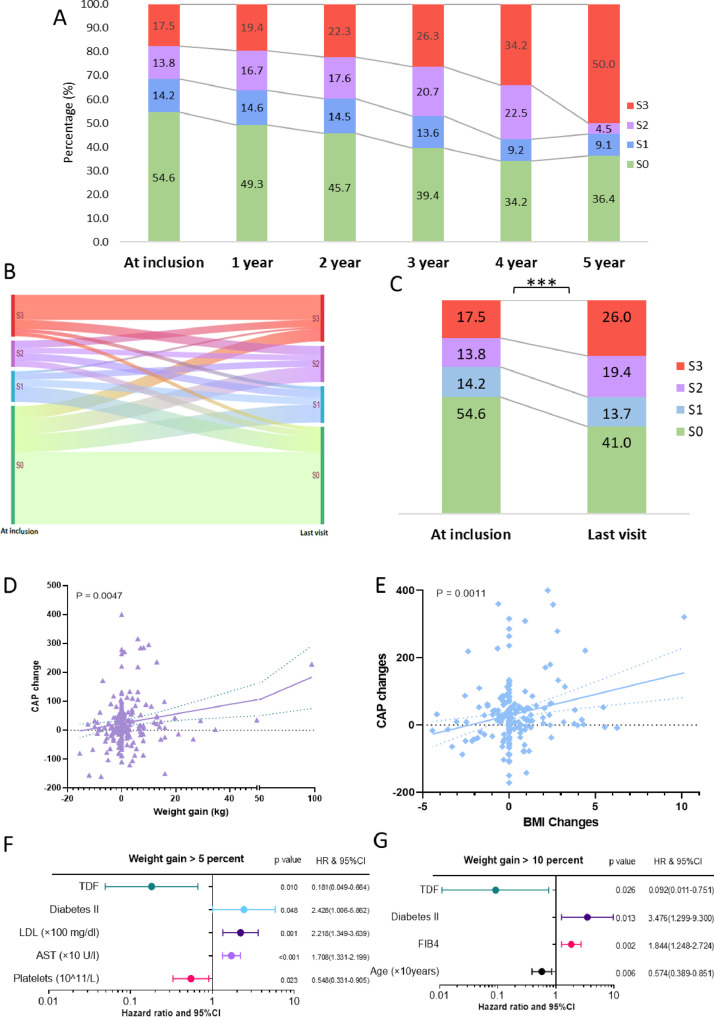
Panel A. Proportion of non-steatosis and different stages of steatosis in each visit; Panel B. River diagram of patients with non-steatosis and different stages of steatosis in first and last visit. Panel C. Percentages of patients with non-steatosis and different stages of steatosis in first and last visit. Comparison of numbers change between two visits were using Chi-square test; ***, *P* < 0·0001; Stages of steatosis Panel A-C: S0 < 238 dB/m, S1: 238–259 dB/m, S2: 260–291 dB/m, S3: >292 dB/m; Panel D. Plot of association of weight gain and CAP changes. P values were calculating using Pearson correlation; Panel E. Plot of association of BMI changes and CAP changes. P values were calculating using Pearson correlation; Panel F. Forrest plot of hazard ratio in multi-variate time-dependent Cox regression of 5 percent weight gain; Panel G. Forrest plot of hazard ratio in multi-variate time-dependent Cox regression of 10 percent weight gain; Abbreviation: ALT, alanine aminotransferase; AST, aspartate aminotransferase; BMI, body mass index; FAST, FibroScan-AST; FIB-4, Fibrosis-4; HR, hazard ratio; LDL, low-density lipoprotein; TDF, tenofovir disoproxil fumarate.

We observed a significant increase in weight and BMI as well as cholesterol, fasting glucose, high-density lipoprotein (HDL), low-density lipoprotein (LDL), AST and albumin during follow-up (Table 1). Scores indicating liver disease progression (FAST Score, FIB-4 score, APRI score and liver stiffness measurements) increased significantly during follow-up with the appearance of steatosis (Table 1). A substantial proportion of patients developed de novo steatosis (20·0%) or progressed to a severer stage of steatosis (7%) during follow-up. Fig. 1A and Supplementary Figure 1A’ description of the proportion of patients, assessed on a yearly basis after five years of follow-up, demonstrate that the severe steatosis of S3 increased from 17·5% to 50·0%. The percentage of the non-steatosis patients decreased from 54·6% to 36·4%. When analyzing the last follow-up, regardless of the observation period, the river diagram (Fig. 1B) shows clearly that steatosis grade increased in nearly 30% of the patients. This shift in the steatosis grade is highly significant in our population (*p*<0·001) (Fig. 1C).

### 3.2 Clinically significant weight gain

Since weight gain is the most intuitive factor of steatosis, we monitored the weight gain in our collective correspondingly. The mean weight of all patients included demonstrated a significant increase during follow-up (77·2 kg ± 14·2 vs. 80·8 kg ± 20·7, *p*<0.001). Since weight gain of >5% and >10% are considered clinically significant, we also determined the corresponding weight gain categories and identified 64 (24·7%) patients, which gained >5% of weight and 34 (13·1%) patients which gained >10% of weight, respectively. Baseline characteristics of each group within the different weight gain categories are displayed in Supplementary Table 4. A statistically significant trend between weight gain (Fig. 1D) and increase of BMI (Fig. 1E) with CAP was found. Significantly more patients who were underweight (BMI<18·5 kg/m^2^) at baseline were found to belong to the category gaining over 10% of weight.

In the Cox regression model with time-dependent covariates to evaluate risk factors for a clinically significant weight gain, neither exposure to INSTIs nor to TAF had a significant effect on weight gain. Interestingly, TDF was significantly associated with a lower risk of >5% of weight gain (Fig. 1F) and >10% of weight gain (Fig. 1G) in both univariable and multivariable analyses, respectively. Additional risk factors for weight gain >5% were diabetes mellitus type 2, higher LDL-cholesterol and AST values, in contrast to platelet values, which were associated with a lower risk. A weight gain >10% was more likely in younger patients with higher FIB4 scores and diagnosis of type 2 diabetes mellitus.

### 3.3 De novo development and progression of hepatic steatosis

To dissect the effect of different cART drugs related to weight gain, we analysed the development and progression of steatosis according to the specific treatment types (Table 2). Mean exposure time to TDF, TAF or INSTI was 33·4 ± 12·7 months, 16·4 ± 7·9 months and 31·3 ± 16·6 months, respectively.

**Table 2:** Comparison of the characteristics between the visit exposure to respective treatment and last visit in each subgroup.

Characteristics	TDF (*n* = 269)			TAF (*n* = 131)			INSTI (*n* = 118)		
	At exposure	Last Visit	*P* value	At exposure	Last Visit	*P* value	At exposure	Last Visit	*P* value
Exposure time, months	··	33·4 ± 12·7	··	··	16·4 ± 7·9	··	··	31·3 ± 16·6	··
Male	211 (78·4)	··	··	109 (83·2)	··	··	94 (79·7)	··	··
Age, years	45·1 ± 11·5	··	··	47·7 ± 11·3	··	··	48·1 ± 12·0	··	··
Weight, kg Weight gain, kg	76·8 ± 14·3 ··	78·7 ± 16·9 1·9 ± 9·8	0·006 ··	79·6 ± 18·2 ··	83·2 ± 24·2 3·6 ± 17·9	0·030 ··	79·3 ± 12·8 ··	82·1 ± 17·1 2·8 ± 13·6	0·070 ··
BMI, kg/m^2^	24·6 ± 4·1	25·2 ± 4·6	0·002	25·5 ± 5·1	26·7 ± 7·3	0·026	25·4 ± 3·7	26·3 ± 5·5	0·066
**Elastography or non-invasive tests**									
CAP, dB/m	233·1 ± 55·7	244·4 ± 55·6	0·001	258·6 ± 59·9	255·8 ± 62·0	0·577	255·3 ± 59·0	253·8 ± 64·8	0·805
Liver stiffness, kPa	5 ± 1·7	5·3 ± 1·9	0·016	5·4 ± 2·5	5·5 ± 3·7	0·565	5·9 ± 3·4	5·9 ± 3·1	0·872
FIB-4 score	0·9 ± 0·4	1 ± 0·5	<0·001	1·0 ± 0·6	1·3 ± 0·8	<0·001	1·0 ± 0·6	1·4 ± 0·8	0·000
APRI score	0·3 ± 0·2	0·3 ± 0·3	0·471	0·4 ± 0·4	0·4 ± 0·3	0·070	0·4 ± 0·3	0·4 ± 0·4	0·047
FAST score	0·1 ± 0·1	0·1 ± 0·1	0·082	0·1 ± 0·2	0·2 ± 0·1	0·045	0·2 ± 0·2	0·2 ± 0·2	0·039
**Laboratory tests**									
Cholesterol, mg/dl	194·0 ± 42·0	202·6 ± 44·4	0·001	204·5 ± 40·7	205·7 ± 38·1	0·710	191·0 ± 45·5	199·7 ± 41·8	0·044
Triglyceride, mg/dl	169·7 ± 128·9	168 ± 149·3	0·818	161·5 ± 89·1	173·0 ± 120·5	0·155	184·4 ± 110·6	192·8 ± 129·8	0·531
HDL, mg/dl	49·1 ± 18·7	54·9 ± 19·9	0·000	53·5 ± 17·5	51·9 ± 16·5	0·188	44·6 ± 14·6	49·0 ± 17·8	0·005
LDL, mg/dl	121·3 ± 33·3	127·4 ± 33·1	0·002	127·7 ± 32·4	144·1 ± 97·0	0·047	118 ± 34·6	140·7 ± 114·5	0·051
AST, U/I	25·3 ± 15·5	25·9 ± 15·1	0·648	25·8 ± 17·9	29·9 ± 16·2	0·015	26·5 ± 15·6	31·8 ± 23·8	0·076
ALT, U/l	36·3 ± 19·2	34·7 ± 22·5	0·321	34·8 ± 21·4	31·5 ± 19·5	0·079	39 ± 24·3	35·3 ± 26·3	0·241
yGT, U/l	55·5 ± 45·9	46·6 ± 36·1	0·001	48·2 ± 40·6	46·6 ± 93·8	0·845	56·2 ± 55·5	45·6 ± 38·9	0·022
Total Bilirubin, mg/dl	0·6 ± 0·5	0·6 ± 0·5	0·828	0·6 ± 0·7	0·6 ± 0·7	0·273	0·6 ± 0·8	0·5 ± 0·3	0·098
Platelets, G/l	232·6 ± 58·1	234·6 ± 60·7	0·478	233·0 ± 61·6	229·4 ± 58·2	0·303	225·6 ± 59·5	226·1 ± 59·8	0·923
Creatinine, mg/dl	0·9 ± 0·2	0·9 ± 0·2	0·073	1·0 ± 0·2	1·0 ± 0·2	0·293	1·0 ± 0·2	1·0 ± 0·2	0·232
Albumin, g/dL	44·3 ± 3·9	45·3 ± 5·7	0·018	45·6 ± 3·9	46·6 ± 2·9	0·001	43·6 ± 4·1	44·8 ± 5·9	0·031
HBA1c,%	5·5 ± 1	5·5 ± 0·9	0·905	5·5 ± 0·9	5·5 ± 1·1	0·401	5·7 ± 1·4	5·8 ± 1·3	0·094
Glucose, mmol/l	94·3 ± 28·4	95·3 ± 25·9	0·481	95·4 ± 22·1	101·2 ± 30·9	0·041	102·3 ± 39	107·0 ± 39·9	0·198
CRP, mg/L	4·2 ± 5·9	3·7 ± 6·6	0·547	3·3 ± 4·6	4·6 ± 11·9	0·230	5·2 ± 11·1	5·0 ± 9·8	0·930

Comparison between two visits in each subgroup were using paired t-test.

Abbreviations: ALT, alanine aminotransferase; APRI, AST to platelet ratio index; AST, aspartate aminotransferase; BMI, body mass index; CAP, controlled attenuation parameter; cART, antiretroviral therapy; CRP, C reactive protein; FAST, FibroScan-AST; FIB-4, Fibrosis-4; HBA1c, hemoglobin A1c; HDL, high-density lipoprotein; INSTI, integrase strand transfer inhibitor; LDL, low-density lipoprotein; TDF, tenofovir disoproxil fumarate.

**Fig. 2:**
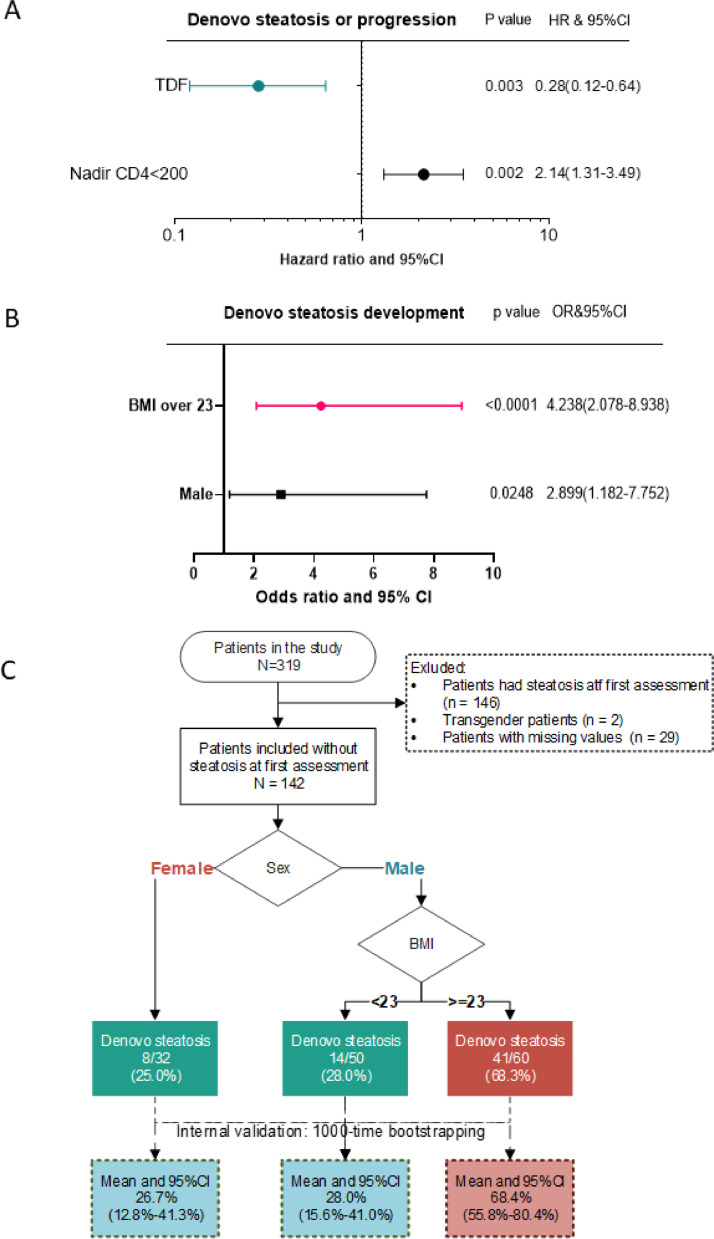
Panel A. Forrest plot of hazard ratio in multi-variate time-dependent Cox regression of de novo steatosis or steatosis progression. Panel B. Forrest plot of odds ratio in multi-variate logistic regression of de novo steatosis in patients without steatosis at baseline; Panel C. Decision tree of de novo steatosis risk among the patients without steatosis at baseline, and mean risk of de novo steatosis with 95% CI among internal validation using 1000-time bootstrapping; Abbreviations: CI, confidence interval.

We observed significant weight gain during the exposure time for those being exposed to TDF and TAF (Table 2). Supplementary Table 5 shows that, regardless of clinically significant weight gain or not, patients with TDF treatment had significant CAP value reduction comparing between the last and the first measurement. Furthermore, in the multivariate Cox regression, the use of TDF was independently associated with a lower risk of development or progression of steatosis. A nadir CD4 < 200/µl was associated with increased risk of steatosis (Fig. 2A).

### 3.4 De novo development of steatosis and decision tree

In order to establish a tool to stratify the risk of steatosis development, we performed a decision tree analysis. Overall, 48·5% (146/301) of patients with valid CAP measurements had steatosis at baseline. Mean follow up time was 41·5 ± 14·8 months (range: 9 - 62months) in those without steatosis and 44·5 ± 13·3 (range: 9 – 63 months; *p* = 0·08) months in patients with steatosis at baseline. Patients without steatosis were significantly younger (42·6 ± 10·9 years vs 48·9 ± 10·7 years; *p*<0·001), leaner (BMI 23·1 ± 2·7 vs 26·3 ± 3·9 *p*<0·001; weight 71·4 ± 11·5 vs 81·9 ± 12·8 *p*<0·001) and displayed a better metabolic profile than patients with steatosis already present at baseline (Supplementary Table 6). HIV RNA was undetectable in 116/141 (82·3%) patients without steatosis at first measurement comparing to 119/132 (90·4%; *p* = 0·04) patients with steatosis.

We further evaluated those patients who developed steatosis during the observational period and compared to those who did not develop steatosis. Overall, 44 0·5% (69) patients developed steatosis. Mean age of these patients was 44·1 ± 11·5 years. Predominantly males developed steatosis (86% vs 69%, *p* = 0·01). Although individuals who developed steatosis had a higher BMI at baseline compared to those who did not develop steatosis (23·9 ± 2·4 vs 22·4 ± 2·8, *p*<0·001), both groups had a BMI within the normal range. All baseline characteristics of both subgroups are summarized in Supplementary Table 7.

In logistic regression model and ROC curve to establish the predictive model and optimal cut-off of the risk factor for de novo steatosis, we found that a BMI of >23 kg/m^2^ for males is significantly associated with development of de novo steatosis. For this cut-off of BMI > 23 kg/m^2^, the specificity is 66·0% and the sensitivity is 81·5%. The area under the ROC curve of the combination model of sex and BMI is 0·7126 (Supplementary Figure 1B), and the calibration plot is shown in Supplementary Figure 1C.

Since BMI > 23 kg/m^2^ and male sex were found to be significantly associated with de novo steatosis development (Fig. 2B), a decision tree was built in Fig. 2C to stratify two groups of patients with different risks for de novo steatosis development. Among patients without steatosis at baseline, 68·3% of male patients with BMI over 23 kg/m^2^ developed steatosis, with only around 25% in female patients. This simple decision tree can stratify patients with significant risk (more than two-third) from patients with low risk (one-fourth) for development of steatosis using only sex and BMI. We further performed a 1000-time bootstrapping in patients without steatosis at baseline for internal validation. Similar mean proportion of de novo steatosis was found in each group stratified by the cut-off value, of which, male patients with BMI over 23 kg/m2 have the highest proportion of de novo steatosis with 68.4%. (Fig. 2C)

**Fig. 3:**
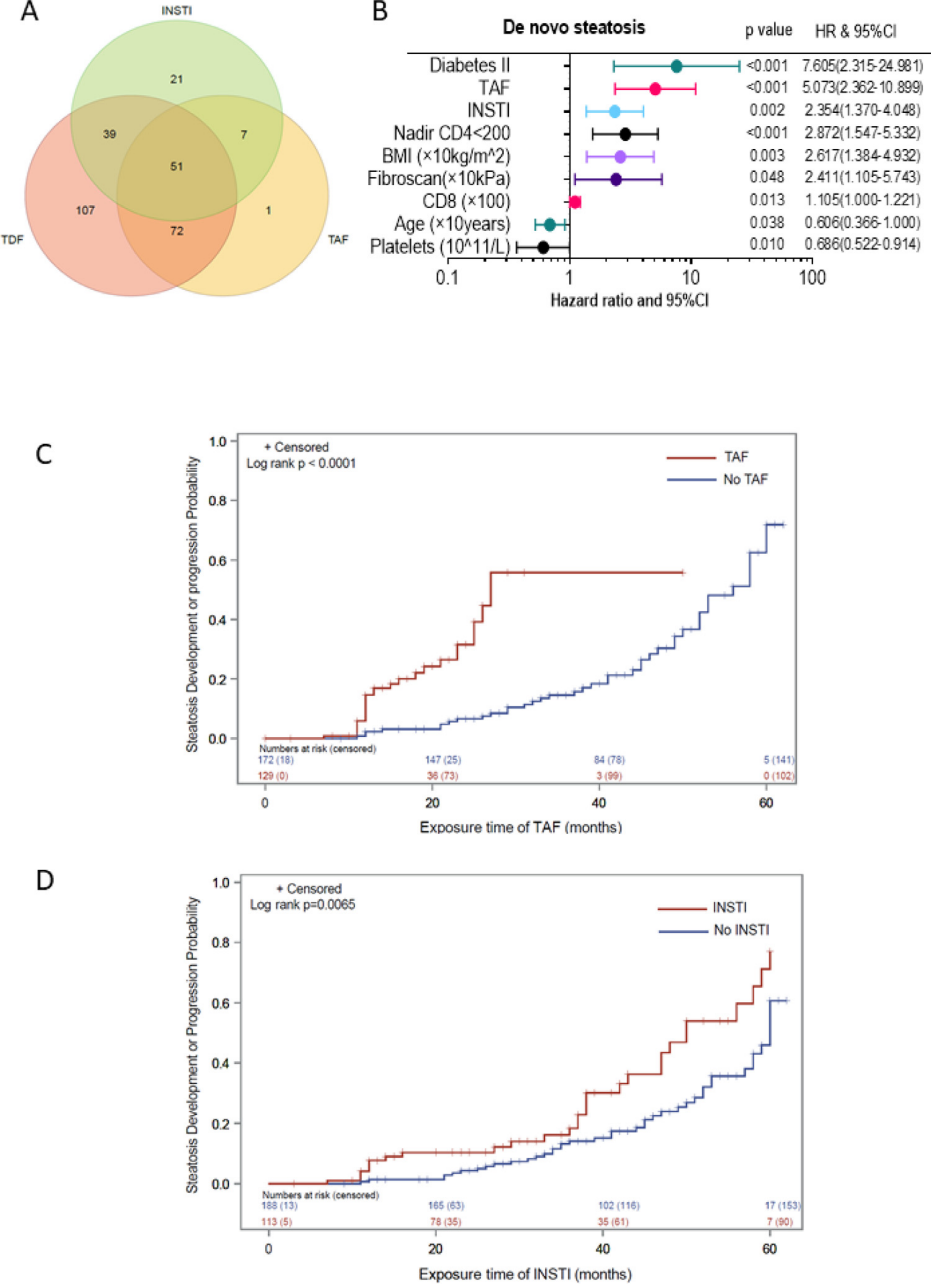
Panel A. Venn diagram of number of patients treated with INSTI, TDF and/or TAF. Panel B. Forrest plot of hazard ratio in multi-variate time-dependent Cox regression of de novo steatosis. Panel C. Cumulative incidence of CAP progression in HIV patients compared between patients treated with TAF or not. P value was calculated by log rank test; Panel D. Cumulative incidence of CAP progression in HIV patients compared between patients treated with INSTI or not. P value was calculated by log rank test. Abbreviation: BMI, body mass index; TDF, tenofovir disoproxil fumarate; INSTI, integrase strand transfer inhibitors; TAF, tenofovir-alafenamid.

From all patients included, 298 (93·4%) patients were treated with TDF, TAF and/or INSTI (Fig. 3A). To search for further factors which may be related to de novo steatosis, we performed a Cox regression analysis for determination of risk factors for de novo development of steatosis (Fig. 3B). Presence of diabetes mellitus, administration of TAF and INSTIs were independently and significantly associated with the development of steatosis. Nadir CD4 cell count < 200/µl, higher BMI, higher liver stiffness, higher CD8+ cell counts, lower platelets and younger age were also independent risk factors of de novo steatosis (Fig. 2D).

Interestingly, patients under TAF based cART developed or progressed steatosis significantly faster than patients without (*p*<0·0001) (Fig. 3C). Similarly, also in patients treated with INSTI showed a significantly faster development or progression of steatosis than patients without INSTI (*p* = 0.006) (Fig. 3D).

### 3.5 Risk for progression to NASH

After evaluating steatosis, we assessed the FAST score to evaluate the risk of progression towards NASH in our patients’ population [17]. Baseline FAST scores showed a statistically strong correlation with baseline FIB4 scores (R: 0·529; *p*<0·001), APRI scores (R: 0·758; <0·001) and also showed a statistically significant correlation with CAP values (R: 0·371; *p*<0·001).

**Fig. 4:**
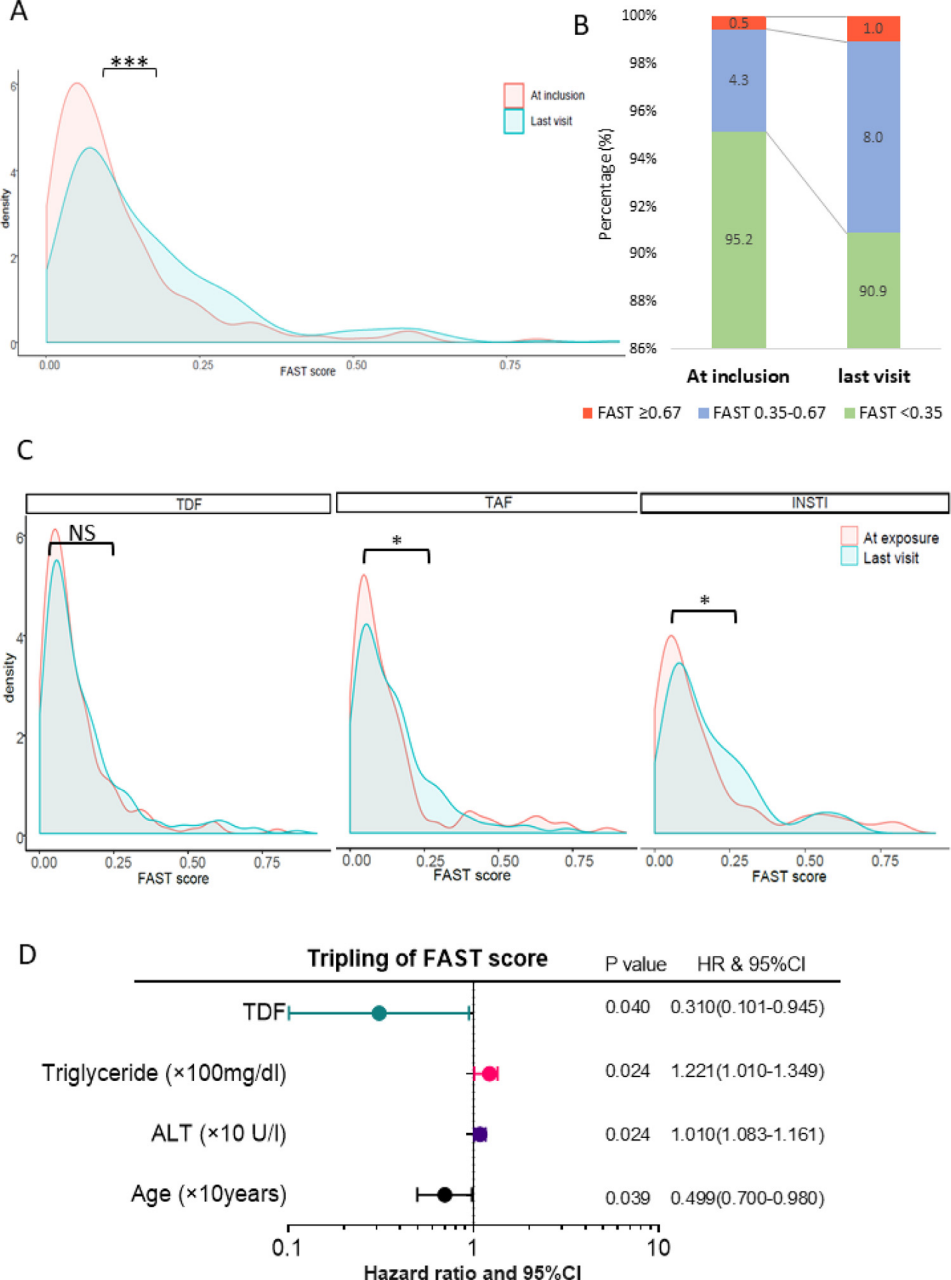
Panel A. Density curve of FAST score at inclusion and last visit. Comparison between two visits were using paired t-test. ***, *P* < 0·0001; Panel B. Density curve of FAST score at inclusion and last visit in patients with TDF, TAF and INSTI. Comparison between two visits were using paired t-test. *, *P* < 0·05; NS, not significant; Panel C. Forrest plot of hazard ratio in multi-variate time-dependent Cox regression of tripling FAST score increase. Abbreviation: ALT, alanine aminotransferase; FAST, FibroScan-AST; HR, hazard ratio; TAF, tenofovir-alafenamid; TDF, tenofovir disoproxil fumarate; INSTI, integrase strand transfer inhibitors.

In our patients, there was a significant increase of FAST score from last visit compared to baseline as shown by the shift towards higher density in higher FAST scores (Fig. 4A). The changes in FAST scores in each group over the observation period are shown in Fig. 4B, which confirmed this trend. Interestingly, in the subgroup analysis, FAST score increased significantly only in patients treated with INSTI and TAF (Fig. 4C). Moreover, administration of TDF was associated with lower risk of a tripling FAST score increase, underlining the protective effect of TDF towards NASH (Fig. 4D). In Cox regression analysis, other variables associated with FAST score progression were higher triglycerides, higher ALT and younger age.

## 4 Discussion

Our prospective study conducted in a unique and large cohort of monoinfected HIV patients followed annually for up to five years, clearly demonstrates that steatosis develops in almost half of our patients over time. A particular high risk for steatosis development was noted in males with a non-obese BMI of >23 kg/m^2^ at baseline. Moreover, development and progression of steatosis, as well as the risk for progression towards NASH is more pronounced in patients receiving INSTI or TAF but might be decreased in patients receiving TDF.

Hepatic steatosis is a hallmark of NAFLD and NASH. In our study, almost 50% of patients had steatosis. This is slightly higher than what has been shown by previous studies [2,26]. However, these studies also confirm the importance of hepatic steatosis in this population. Our study is unique, especially under consideration of the long follow up of up to five years, which strengthens the certainty of steatosis progression using intra-individual comparisons. The collection of data over an extended period of time in a clinical setting nicely reflects clinical reality, which in many cases is not well depicted within randomised control trials. Such an approach could also be utilized in HIV-negative pre-exposure prophylaxis (PrEP) users or HIV/HCV coinfected patients in order to examine specific risk factors in these patient groups with regard to risk for development of fatty liver disease but also development of diabetes related to specific drugs in these patients. Conducting such a study in HIV negative PrEP users would also provide an interesting control group to examine the influence of TAF or TDF on the development of NAFLD and body weight changes, respectively. Unfortunately, to our knowledge, so far only weight data is being collected in these trials.

In the HIV negative population, BMI, age and metabolic syndrome are associated with an increased risk to develop hepatic steatosis [2,27,28]. However, in previous studies, not many other factors have been reported to predict steatosis in PLHIV [21,29].

Our data advocate for weight monitoring, since weight gain and increase of BMI are significantly correlated with increase of steatosis measured by CAP. Interestingly, both, development of steatosis and weight gain were severely influenced by choice of cART drugs. In particular, non-boosted INSTI and TAF have been reported to be associated with weight gain in first-line treatment studies as well as in switch trials [20,21,23,[30], [31], [32]]. Recent findings from the ADVANCE study clearly show a relevant weight gain with dolutegravir (DTG) at week 96 with the largest increase in patients initiating combination therapy with TAF/Emtricitabine (FTC) and DTG [32]. Strikingly, in our study, INSTI and TAF also had a high risk for development of steatosis and were associated with a faster progression of steatosis grade over time. These data are well in line with previously published data, which showed that both substances were associated with a significant weight gain after initiation of therapy [20,22,23,31,33]. However, we cannot exclude an effect of previous therapies which may influence our results. [30] Yet, our study is the first to demonstrate a clear link between INSTI and/or TAF use and occurrence of hepatic steatosis in the context of increased body weight gain, which are relevant events in the development of NAFLD.

Interestingly, we found TDF to be protective against weight gain and therefore might be protective for development of steatosis as well, as indicated by our analysis. Indeed, a weight suppressive effect of TDF has already been postulated in other controlled ART trials. [34] TDF has also been shown to cause a moderate decrease in blood lipids and weight reduction in PrEP-studies [35], confirming the protective effect of TDF as reported in our study. While the mechanism by which TDF might protect from development or progression of steatosis have to be further investigated to exclude a solely effect of weight suppression, the beforehand reported weight suppressive effect of TDF is supported by our results, since patients under TDF were protected against clinically significant weight gain of more than 5% or 10%, respectively. Further support comes from ART studies which also highlight the weight suppressive effect of TDF which is unmasked or reverted after switching from TDF to TAF [36]. In fact, Mayer et al. described quite recently, that the average weight gain with TAF in HIV negative PrEP users was not different from the annually expected weight gain in the general population [37]. In contrast, the results of the CoRIS cohort study support our findings that TAF itself is associated with weight gain by showing significantly greater weight gain with TAF in patients newly commencing treatment with TAF or TDF. [31] Various other explanations for different observations in weight changes related to cART include a return-to-health effect in the first years after therapy initiation, altered regulation of appetite, and interactions with the MC4 receptor [19,38,39].

Still the question remains, how clinically relevant are the changes observed in our cohort in up to five years of follow-up. In order to address this, we first used the recently validated FAST score [17]. This score has been validated in several cohorts, but so far has not been used in HIV positive patients. Our study demonstrated that the FAST score was strongly correlated with FIB-4, APRI and CAP values, underlining the validity in our patient cohort. Similar to weight gain and development or progression of steatosis grade, we observed a strong effect of individual cART drugs on FAST score increase. Indeed, as an endpoint we chose a tripling of the FAST score, to assess the risk for clinically relevant NASH development. Most interestingly, TDF prevented the increase in FAST scores, while TAF and INSTI were shown to increase FAST scores during the exposure time on the specific drugs, respectively. We believe that our findings can contribute to the improvement of screening and prevention measures as well as the management of NAFLD in HIV positive patients. Our study provides an impulse for a critical re-evaluation of the already recommended algorithms in the guidelines of the European AIDS Clinical Society. In addition, we believe, that NAFLD in PLHIV, as a special patient population at risk, should be included in future reviews on natural history, epidemiological patterns and therapeutic strategies of NAFLD. [40] There are, however, some limitations of our work. Our study population consisted mainly of white Caucasian men and only few black patients. We did not focus on lifestyle interventions as well as genetic polymorphisms known to be associated with NAFLD. Yet our study has a very long follow-up and allowed for intra-individual comparisons which might outweigh a potential bias.

To conclude, this study clearly indicates that weight gain and development or progression of hepatic steatosis are linked and that INSTIs as well as TAF might impact this progression considerably. Nearly half of the patients developed de novo steatosis in the observational period, even with non-obese BMI at baseline. Our findings point towards a protective role of TDF on weight changes and therefore or maybe even independently on development or progression of steatosis and on the risk for progression towards NASH. By contrast, TAF and INSTI known to increase body weight, promoted the development or progression of hepatic steatosis, and increased the risk of NASH. Our study allocates for hepatic phenotyping of patients on these drugs and male patients with BMI more than 23 kg/m^2^ during follow-up.

## Contributors

JKR, JT, CB and CSZ had the initial idea (conceptualisation of the study), JKR, CB, CSZ, KvB, LD, JB and JCW collected the data, JT, JKR, WG and JB accessed the raw data and are responsible for the raw data, JT, WG and JB analysed the data, JB wrote the first draft of the manuscript. All authors contributed to study design, commenting on drafts and revisions.

## Data sharing statement

The data supporting the findings of this study, deidentified participant data or related documents as the informed consent form can be made available from the corresponding author JT upon reasonable request. Data identifying any study participant will not be made available. Contact information for JT are included on the title page. JT, JKR, WG and JB have access to the raw data and show responsibility for these. The datasets are no subjects to restrictions or embargo.

## Funding

There was no additional funding received for this project. All funders mentioned in the ‘declaration of interests’ section had no influence on study design, data collection and analysis, decision to publish, or preparation of the manuscript.

## Declaration of Competing Interest

Jonel Trebicka is supported by grants from the Deutsche Forschungsgemeinschaft (SFB TRR57 to P18, CRC 1382AO9), European Union's Horizon 2020 Research and Innovation Programme (Galaxy, No. 668,031 and, MICROB-PREDICT, No. 825,694 and DECISION, No.847949), and Societal Challenges - Health, Demographic Change and Wellbeing (No. 731,875), and Cellex Foundation (PREDICT).

Jürgen Rockstroh has received honoraria for consulting or speaking at educational events from Abivax, Galapagos, Gilead, Merck, Janssen, Theratechnologies and ViiV.

Jenny Bischoff is supported by a scholarship of the BONFOR research support program for young scientists at the Rheinische Friedrich-Wilhelms-Universität (BONFOR Funding Instrument 1, Type A; Application number: 2020–1A-08).

Christoph Boesecke has received honoraria for lectures from Abbvie, Gilead, ViiV, Janssen and MSD and was supported by a Grant from Hector-Stiftung for another HIV Study.

Jan-Christian Wasmuth has received a travel grant from Gilead to attend a meeting.

Wenyi Gu, Leona Dold, Carolynne Schwarze-Zander and Kathrin van Bremen have nothing to declare.

All funders had no influence on study design, data collection and analysis, decision to publish, or preparation of the manuscript.

## Appendix Supplementary materials

Image, application 1
