# Supplementary material for: Stratifying the risk of NAFLD in patients with HIV under combination antiretroviral therapy (cART)
Source: eClinicalMedicine. 2021 Sep 5;40:101116. doi: 10.1016/j.eclinm.2021.101116 (PMC8427211; doi:10.1016/j.eclinm.2021.101116)
Supplement: Supplementary file 1 [file mmc1.docx]

**Caption for supplementary material**

**Manuscript title: Stratifying the risk of NAFLD in patients with HIV under combination antiretroviral therapy (cART)**

Jenny Bischoff MD^1^*, Wenyi Gu^2^*, Carolynne Schwarze-Zander^1,3^, Christoph Boesecke^1,3,^ Jan-Christian Wasmuth^1,3^, Kathrin van Bremen^1^, Leona Dold^1,3^, Jürgen K Rockstroh^1,3#^ Jonel Trebicka^2 #^

**Supplementary Table 1:** Numbers of valid data of each parameter on baseline and last visit

**Supplementary Table 2:** Number of valid data of each parameter on baseline in patients without steatosis and developed de novo steatosis.

**Supplementary Table 3:** Univariate Cox regression of different chronic diseases on denovo steatosis or progression, and on tripling of FAST score

**Supplementary Table 4:** Comparison steatosis and no steatosis baseline

**Supplementary Table 5:** Subgroup analysis in patients with or without clinically significant weight gain of changes of CAP value and FAST scores in patients with or without TDF treatment

**Supplementary Table 6:** Comparison of data on baseline between patients without steatosis and patients developed de novo steatosis

**Supplementary Table 7:** Comparison baseline characteristics between the group with or without clinically significant weight gain

**Supplementary Figure 1:** River diagram of number of patients without steatosis or different grades of steatosis at each visit; area under the curve of logistic regression model of de novo steatosis combined with sex and BMI; and calibration plot of logistic regression model of de novo steatosis combined with sex and BMI
